# Sex differences in the intellectual functioning of early school-aged children in rural China

**DOI:** 10.1186/s12889-016-2956-6

**Published:** 2016-03-29

**Authors:** Chao Li, Ni Zhu, Lingxia Zeng, Shaonong Dang, Jing Zhou, Yijun Kang, Yang Yang, Hong Yan

**Affiliations:** School of Public Health, Xi’an Jiaotong University Health Science Center, No.76 West Yanta Road, Xi’an, Shaanxi 710061 PR China; Department of Health Information, Shaanxi Provincial Centre for Disease Control and Prevention, Xi’an, PR China; Department of Planned Immunization, Xi’an Center for Disease Control and Prevention, Xi’an, PR China; Nutrition and Food Safety Engineering Research Center of Shaanxi Province, Xi’an, PR China

## Abstract

**Background:**

Gender disparities in China are concentrated in poor rural areas and among poor households. The difference in intelligence between boys and girls is less clear in rural China. The purpose of this paper was to assess sex differences in the intellectual function of early school-aged children in rural China.

**Methods:**

One thousand seven hundred forty four early school-aged offspring of women who had participated in a prenatal supplementation trial with different combinations of micronutrients and continued to reside in two rural counties in China were followed. We measured their Full-Scale IQ (FSIQ), Verbal Comprehension Index (VCI), Working Memory Index (WMI), Perceptual Reasoning Index (PRI) and Processing Speed Index (PSI) using the Wechsler Intelligence Scale for Children (WISC-IV). Multilevel analyses were used to assess sex differences in intellectual functioning in 7-10-year-old children in rural China.

**Results:**

Boys’ adjusted mean FSIQ score was 0.97 points higher (95 % CI: -2.22 − 0.28) than that of girls. Girls obtained higher mean WMI and PSI scores, with 1.32 points (95 % CI: 0.14 − 2.51) and 3.10 points (95 % CI: 1.82–4.38) higher adjusted means, respectively. Boys’ adjusted mean VCI and PRI scores were significantly higher than those of girls, and the mean differences were 2.44 points (95 % CI: 0.95 − 3.94) and 3.68 points (95 % CI: 2.36 − 5.01), respectively.

**Conclusions:**

There is no evidence to suggest sex differences in the general intelligence of early school-aged children in rural China. However, a difference in general intelligence between 10-year-old boys and girls was evident. Girls and boys in rural China tended to show different specific cognitive abilities.

## Background

Sex differences in general intelligence has been a subject of lively debate in recent years [1]. Since the early years of the twentieth century, it has been consistently asserted that there is no sex difference in average general intelligence, defined as the sum of cognitive abilities measured by individuals’ IQ scores obtained on tests [2]. This consensus has been challenged by a developmental theory of sex differences in intelligence. Lynn contended that there is virtually no sex difference in average intelligence between the ages of 5 and 10 years; that between the ages of 11 and 14 years, girls have a small IQ advantage of approximately 1 IQ point because they mature earlier; and that from the age of 15 to 16 years, boys develop a small IQ advantage of approximately 1 IQ point, which increases in later adolescence and reaches approximately 4 IQ points in adulthood [3, 4]. Many studies have confirmed the developmental theory of Lynn. Evidence from the United States showed a male advantage of 2.8 IQ points among 22–23-year-old whites [5]. Two studies conducted in Germany showed a male advantage of 6.0 IQ points in a sample of 34-year-olds and 9.7 IQ points in a sample of 16-year-olds [6, 7].

China adheres to a Confucian gender ideology, which emphasises women’s traditional roles as wives, mothers, and caretakers of the family. Although the country has rapidly developed in recent years, this development did not eliminate “male superiority” or individuals’ preference for sons. In Western societies, both male and female children are provided with equal educational opportunities by their parents, whereas in rural China, females experience discrimination by not only society but also their parents [8]. One study revealed that males obtained significantly higher Full-Scale IQ (FSIQ) scores than females in a Chinese urban preschool sample [9]; however, few studies assessed the sex difference in intelligence in rural China. The intelligence difference between early school-aged boys and girls in rural China is less clear. Therefore, in this paper, we aim to assess sex differences in the intellectual functioning of early school-aged children in rural China, where sons are preferred.

## Methods

### Study design

This study focused on follow-up assessments of early school-aged children’s mental and psychomotor development. These children were born to women involved in a double-blind, cluster randomised controlled trial of prenatal micronutrient supplementation that aimed to determine the effect of micronutrient supplementation during pregnancy on birth weight. The details of the double-blind, cluster randomised controlled trial of prenatal supplementation with different combinations of micronutrients are described elsewhere [10–12]. Briefly, this trial was conducted in Changwu and Bing counties in Shaanxi Province of northwest China from 2002 to 2006. We allocated the same treatment to all pregnant women in the same village, and the randomisation of villages was stratified according to county and township to ensure geographic balance.

### Setting

Changwu and Bing counties are situated on the western part of Shaanxi Province. The total area of the two counties is approximately 1752 square kilometres. In 2007, the total population of the two poor, rural counties was approximately 497 thousands, and 90.3 % of the residents were engaged in agricultural work. The agricultural annual per capita net income was only 780 yuan and 1249 yuan in Changwu and Bing counties, respectively. The two counties were type 4 (poorest) rural counties.

### Participants

From October 2012 to September 2013, the offspring of women who had participated in the prenatal micronutrient supplementation trial and continued to reside in the study area were selected for the present follow-up study. In the present study, we excluded children with birth defects and children who had an illness that affected their intellectual development at the time of the study. Interviews were administered at the local hospital or school in a standardised manner by rigorously trained examiners who had demonstrated high levels of consistency with each other in terms of collecting household information from parents, performing physical measurements and conducting psychological tests with children. The purpose of the study was explained to the participants, and parental written consent and child assent were obtained.

We estimated that a minimum of 284 children (142 children in each group) would be needed to detect a minimum difference of 5 FSIQ points (33.3 % of SD), considered clinically significant [13], between groups with 80 % power and type I error (*α =* 0.05). We followed up a total of 1744 children in the present study. This sample size was able to detect a difference of 2.3 FSIQ points (15.3 % of SD) between groups with 80 % power and type I error (*α =* 0.05).

### Psychological testing

We assessed children’s intellectual function by using the latest version of the Wechsler Intelligence Scale for Children (WISC-IV). The WISC-IV can be administered to children between the ages of 6 and 16 years and has ten core subtests and four supplemental subtests. The ten core subtests are Similarities, Vocabulary, Comprehension, Block Design, Picture Concepts, Matrix Reasoning, Digit Span, Letter-Number Sequence, Coding, and Symbol Search, and the four supplement subtests are Picture Completion, Cancellation, Information and Arithmetic. A total of five composite scores can be derived from the WISC-IV. The WISC-IV generates a FSIQ, which represents overall cognitive ability, and four other composite scores. The four other composite scores are the Verbal Comprehension Index (VCI), which is composed of subtests measuring verbal abilities utilising reasoning, comprehension and conceptualisation; Working Memory Index (WMI), which is composed of subtests measuring attention, concentration and working memory; Perceptual Reasoning Index (PRI), which is composed of subtests measuring perceptual reasoning and organisation; and Processing Speed Index (PSI), which is composed of subtests measuring the speed of mental and graphomotor processing [14].

The WISC-IV has been commercialised in China and established as the Chinese norm. These scales have been translated into Chinese and locally standardised for cultural appropriateness. The reliability and validity of these scales have been tested and shown to be satisfactory [15]. Gender invariance is an essential issue pertaining to the WISC-IV. The analysis of a sample in the United States showed that the two sexes display the same mean WISC-IV subtest and index scores. Thus, the WISC-IV scores of boys and girls can be interpreted in the same way [16].

### Anthropometry and Household information

At the survey site, trained anthropometrists measured children’s height and weight using standard procedures. Interviews were conducted with the parents to collect information about household demographics and socioeconomic status. For children, information about type of school, number of completed and repeated years of schooling and health status in the last two weeks was collected.

### Statistical analysis

All data were checked manually for completeness and were double-entered into a data management system. We conducted range, extremum and logical checks for accuracy.

Girls and boys’ baseline characteristic were compared using analysis of variance or *χ*^2^ tests. Weight-for-age and height-for-age were calculated using the international reference standard [17]. A wealth index was constructed from an inventory of 16 household assets or facilities using a principal component analysis method [18], and this index was used to categorise three groups: the poorest, middle income, and richest households. Because of the multilevel hierarchical structure of the data, the multilevel model approach was selected for this cluster sampling investigation. Finally, a 3-level analysis was developed to compare the FSIQ, VCI, WMI, PRI and PSI, with township as level 3, village as level 2 and individual as level 1. Estimations of the mean difference and 95 % confidence intervals (CIs) were calculated by sex of children. Variables that were unbalanced by sex of children and associated with outcomes were controlled in the adjusted analyses. Age, schooling, gestational age at birth and household wealth index were strongly associated with children’s mental development [19–21]. In addition, mother’s educational level, presence of siblings, stunting or underweight, birth weight and morbidity of cold in the past two weeks differed by sex of children. Therefore, we ran models that adjusted for these variables and type of prenatal micronutrient supplementation as confounders to assess means difference in FSIQ, VCI, WMI, PRI and PSI between girls and boys. Statistical significance was set at *P* value < 0.05 for all statistical tests. Data were analysed using STATA software, version 12.0. The study was approved by the Human Research Ethics Committee of the Xi’an Jiaotong University Health Science Center.

## Results

By September 2013, 1638 of the children had moved out of the study area and 96 had died. Of the remaining 2865 children eligible for inclusion in the present study, 1744 were followed during the study period. Analysis of variance or *χ*2 tests were used to compare boys and girls’ baseline characteristics. The multilevel model approach was selected to compare differences in FSIQ, VCI, WMI, PRI and PSI between boys and girls.

Table 1 shows the baseline characteristics of children and households for boys and girls. The mean age of the investigated children was 8.78 (SD, 0.83) years and did not differ by sex (*P =* 0.096), andmost investigated children were 8 (42.2 %) and 9 years (30.6 %) of age. The majority of children (75.6 %) studied in a township or village school, and the school distribution did not differ by (*P =* 0.096). The proportions of stunting and underweight were 6.3 % and 8.4 % for girls and 3.4 % and 5.3 % for boys, respectively. The proportions of stunting (*P =* 0.004) and underweight (*P =* 0.009) were significant greater among girls. Similarly, more girls (71.1 %) reported lower respiratory tract symptoms in the last two weeks than boys (65.3 %). In general, boys and girls differed with respect to maternal age, presence of siblings, percentage of stunting and underweight, and lower respiratory tract symptoms in the last two weeks. However, the proportions of educational level, occupational classification of parents, household socioeconomic status, prenatal history of pneumonia, prenatal micronutrient supplementation type, gestational age at birth and mean age of children did not differ by sex of children.

**Table 1:** Comparison of the baseline characteristics of children, their mothers and households by sex^a^

	Gender		*P*
	Boys	Girls	
Number of children	1045	699	
Children age			
Mean (SD)	8.81 ± 0.03	8.74 ± 0.03	0.096
7 y	194 (18.6 %)	136 (19.5 %)	0.307
8 y	426 (40.8 %)	310 (44.3 %)	
9 y	334 (31.9 %)	199 (28.5 %)	
10 y	91 (8.7 %)	54 (7.7 %)	
Maternal age (years)			
Mean (SD)	34.44 ± 0.14	33.23 ± 0.16	<0.001
Women’s education			
Primary	389 (37.3 %)	244 (35.0 %)	0.616
Secondary	538 (51.6 %)	371 (53.2 %)	
High school+	116 (11.1 %)	82 (11.8 %)	
Father’s education			
Primary	174 (16.7 %)	114 (16.4 %)	0.943
Secondary	654 (62.7 %)	442 (63.5 %)	
High school+	215 (20.6 %)	140 (20.1 %)	
Women’s occupation			
Farmer	690 (67.5 %)	460 (67.1 %)	0.843
Others	332 (32.5 %)	226 (32.9 %)	
Father’s occupation			
Farmer	380 (37.3 %)	259 (37.6 %)	0.888
Others	640 (62.7 %)	430 (62.4 %)	
Household wealth index			
Poorest-1st tertile	334 (32.0 %)	239 (34.2 %)	0.574
2nd tertile	358 (34.2 %)	226 (32.3 %)	
Richest-3rd tertile	353 (33.8 %)	234 (23.5 %)	
Parity			
0	591 (56.6 %)	508 (72.7 %)	<0.001
1	391 (37.4 %)	162 (23.2 %)	
≥2	63 (6.0 %)	29 (4.1 %)	
School			
Village	341 (32.6 %)	255 (36.5 %)	0.096
Township	431 (41.3 %)	290 (41.5 %)	
County and plus	273 (26.1 %)	154 (22.0 %)	
Birth weight			
Mean (SD)	3.26 ± 0.01	3.11 ± 0.02	<0.001
Gestation at delivery (weeks)			
Mean (SD)	39.81 ± 0.05	39.92 ± 0.07	0.163
Stunted			
Yes	35 (3.4 %)	44 (6.3 %)	0.004
No	1010 (96.6 %)	655 (93.7 %)	
Underweight			
Yes	55 (5.3 %)	59 (8.4 %)	0.009
No	990 (94.7 %)	640 (91.6 %)	
History of pneumonia			
Yes	411 (63.9 %)	290 (65.3 %)	0.636
No	232 (36.1 %)	154 (34.7 %)	
Lower respiratory tract infection			
Yes	664 (65.3 %)	489 (71.1 %)	0.012
No	353 (34.7 %)	199 (28.9 %)	
Treatment group			
Folic acid	355 (34.0 %)	249 (35.6 %)	0.606
Folic acid/Iron	346 (33.1 %)	216 (30.9 %)	
Multi micronutrients	344 (32.9 %)	234 (33.5 %)	

^a^Values are given as Mean ± SD or the percentage of the study population in different supplementation group

Table 2 shows the children’s WISC-IV test scores. The children’s mean FSIQ, VCI, WMI, PRI and PSI scores were 89.48 (SD, 13.34), 87.83 (SD, 15.90), 91.04 (SD, 12.35), 93.12 (SD, 13.89) and 95.68 (SD, 13.34), respectively. Boys exhibited higher mean FSIQ (90.14), VCI (89.17) and PRI (94.87) scores than girls, and girls showed higher WMI (91.67) and PSI (97.35) scores than boys.

**Table 2:** Mean scores on WISC-IV test by sex^a^

	Boys	Girls	Total
FSIQ	90.14 ± 13.35	88.49 ± 13.41	89.48 ± 13.34
VCI	89.17 ± 15.57	85.84 ± 16.18	87.83 ± 15.90
WMI	90.63 ± 12.31	91.67 ± 12.38	91.04 ± 12.35
PRI	94.87 ± 13.60	90.51 ± 13.90	93.12 ± 13.89
PSI	94.56 ± 13.41	97.35 ± 13.06	95.68 ± 13.34

^a^Values are given as Mean ± SD

Boys’ mean FSIQ score, which represents general intelligence, was higher than that of girls, but the difference was not significant. The mean difference in FSIQ was-1.19 points (*P =* 0.056, 95 % CI: -2.41 − 0.03) in the univariate analysis and-0.97 points (*P =* 0.128, 95 % CI: -2.22 − 0.28) after adjusting for the confounders. In the subgroup analysis of different age groups, the results showed that 10-year-old boys exhibited a mean FSIQ score that was 5.29 points (*P =* 0.027, 95 % CI:-9.99 − -0.60) higher than that of their female counterparts in the univariate analysis and 4.91 points (*P =* 0.038, 95 % CI: -9.56 − -0.26) higher in the adjusted analysis. Boys’ mean VCI and PRI scores were significant higher than those of girls. Boys’ unadjusted mean scores were 2.85 points (*P <* 0.001, 95 % CI: 1.41 − 4.30) and 3.95 points (*P <* 0.001, 95 % CI: 2.67 − 5.24) higher. The results were similar after adjusting for confounders. Specifically, the adjusted VCI and PRI mean score differences between boys and girls were 2.44 points (*P =* 0.001, 95 % CI: 0.95 − 3.94) and 3.68 points (*P <* 0.001, 95 % CI: 2.36 − 5.01). Girls exhibited higher WMI and PSI mean scores, and the mean differences were 1.16 points (*P =* 0.048, 95 % CI: 0.01 − 2.30) and 2.91 points (*P <* 0.001, 95 % CI: 1.67 − 4.14), respectively, in the univariate analysis. Adjusting for the confounders did not change the univariate analysis results, as girls’ adjusted WMI and PSI mean scores were 1.32 points (*P =* 0.029, 95 % CI: 0.14 − 2.51) and 3.10 points (*P <* 0.001, 95 % CI: 1.82 − 4.38) higher, respectively, than those of boys. The results of subgroup analysis of different age groups were similar to the results of the total sample. Among the 8-, 9-, and 10-year-old children, boys displayed significantly higher mean VCI and WMI scores, and the unadjusted and adjusted PSI mean score differences between 8-year-old boys and girls were 3.85 points (*P <* 0.001, 95 % CI: 2.41 − 6.46) and 4.43 points (*P <* 0.001, 95 % CI: 2.41 − 6.46), respectively. Among the 7-year-old children, girls’ WMI and PSI mean scores were 2.99 points (*P =* 0.020, 95 % CI: 0.46 − 5.52) and 5.05 points (*P <* 0.001, 95 % CI: 2.32 − 7.77) higher. However, in the adjusted analysis boys obtained a higher mean PRI score (Table 3).

**Table 3:** Comparison of WISC-IV test scores by sex for different age groups^a^

	Girls			
	Unadjusted analysis		Adjusted analysis	
	MD (95 % CI)	*P*	MD (95 % CI)	*P*
Total^b^				
FSIQ	−1.19 (-2.41,0.03)	0.056	−0.97 (-2.22,0.28)	0.128
VCI	−2.85 (-4.30,-1.41)	<0.001	−2.44 (-3.94,-0.95)	0.001
WMI	1.16 (0.01,2.30)	0.048	1.32 (0.14,2.51)	0.029
PRI	−3.95 (-5.24,-2.67)	<0.001	−3.68 (-5.01,-2.36)	<0.001
PSI	2.91 (1.67,4.14)	<0.001	3.10 (1.82,4.38)	<0.001
7 y^c^				
FSIQ	0.88 (-1.76,3.51)	0.513	0.01 (-2.63,2.64)	0.996
VCI	0.24 (-2.71,-3.19)	0.875	0.05 (-2.94,3.04)	0.974
WMI	3.45 (0.94,5.96)	0.007	2.99 (0.46,5.52)	0.020
PRI	−2.75 (-5.89,0.39)	0.086	−3.85 (-7.03,-0.67)	0.018
PSI	5.24 (2.51,7.97)	<0.001	5.05 (2.32,7.77)	<0.001
8 y^c^				
FSIQ	−1.19 (-3.10,0.72)	0.223	−0.12 (-2.02,1.79)	0.906
VCI	−3.14 (-5.40,-0.88)	0.006	−2.39 (-4.67,-0.11)	0.040
WMI	0.79 (-1.02,2.60)	0.393	1.55 (-0.28,3.39)	0.097
PRI	−4.45 (-6.42,-2.89)	<0.001	−3.09 (-5.09,-1.10)	0.002
PSI	3.85 (1.83,5.86)	<0.001	4.43 (2.41,6.46)	<0.001
9 y^c^				
FSIQ	−1.69 (-3.93,0.55)	0.140	−1.92 (-4.12,0.29)	0.088
VCI	−3.84 (-6.56,-1.12)	0.006	−4.56 (-7.44,-1.67)	0.002
WMI	1.11 (-0.93,3.15)	0.285	0.78 (-1.39,2.96)	0.481
PRI	−2.71 (-4.95,-0.48)	0.017	−3.57 (-5.90,-1.23)	0.003
PSI	0.24 (-1.87,2.35)	0.824	0.07 (-2.02,2.16)	0.947
10 y^c^				
FSIQ	−5.29 (-9.99,-0.60)	0.027	−4.91 (-9.56,-0.26)	0.038
VCI	−6.38 (-11.78,-0.98)	0.021	−6.34 (-11.99,-0.70)	0.028
WMI	−1.66 (-6.17,2.84)	0.469	−2.26 (-6.76,2.25)	0.327
PRI	−8.63 (-13.55,-3.71)	0.001	−6.79 (-11.81,-1.76)	0.008
PSI	1.58 (-2.79,5.95)	0.479	3.14 (-1.29,7.58)	0.165

*Abbreviation:* *MD* mean difference, *CI* confidence interval, *FSIQ* full-scale intelligence quotient, *VCI* verbal comprehension index, *WMI* working memory index, *PRI* perceptual reasoning index, *PSI* processing speed index

^a^Multilevel models were used to assess the WISC-IV test scoresdifferences between boys and girls in early school aged children, with township to level 3, village to level 2, and individual to level 1

^b^ *P* value adjusted for the age of children, household wealth index, maternal age, type of prenatal micronutrient supplementation, number of children in home, child school level, birth weight, gestational weeks, whether stunted or underweight children and lower respiratory tract infection in the last two weeks

^c^ *P* value adjusted for the household wealth index, maternal age, type of prenatal micronutrient supplementation, number of children in home, child school level, birth weight, gestational weeks, whether stunted or underweight children and lower respiratory tract infection in the last two weeks

## Discussion

The major findings in this sample from two rural counties in China are that there was no overall sex difference in general intelligence, but there was a difference in general intelligence between boys and girls at the age of 10 years. Specifically, girls displayed lower FSIQ scores. Girls and boys tended to exhibit different specific cognitive abilities, and this tendency was consistent across the ages of 7 to 10 years.

The sex difference in general intelligence has been lively discussed in recent years. The findings on this topic are inconsistent. Some researchers report a male advantage [5–7], others report no differences between the sexes [22, 23], and still others suggest a female advantage [24]. Lynn noted that different studies examine participants of different ages and argued that the findings are, therefore, not comparable. However, Lynn’s developmental theory contends that there is virtually no sex difference in the average intelligence of children aged 5-10 years [3]. The present study results confirm the developmental theory of sex differences in intelligence, showing that there is virtually no sex difference in average intelligence between boys and girls aged 7–10 years. However, at the age of 10 years, a difference in general intelligence between boys and girls was evident. This finding is consistent with the results from a previous study examining an urban Chinese preschool sample, which showed that males obtained significantly higher FSIQ scores than females (2.1 points). In the present study, the sex difference found in the FSIQ scores (4.9 points) of 10-year-old children in rural China was greater than the difference found in the urban preschool sample [9]. This difference may be due to the traditional preference for males that continues to exist in China today despite Westernisation during the late 20th century. This male preference is more prevalent in rural China [25]. Therefore, Chinese boys may receive more cognitive enrichment, including early educational exposure and nutritional advantages, during the prenatal and early childhood periods, and previous studies have shown that early nutritional factors have long-term effects on children’s cognitive development [26].

In the present study, we found that intellectual functioning differed by sex. Girls obtained higher WMI and PSI scores, and boys obtained higher VCI and PRI scores. We also obtained similar results in the subgroup analysis of different age groups. This finding is consistent with previous observations of sex differences [9, 27]. Previous studies revealed that human males (not only in different periods of life (preschool age, school age, adult) but also in different countries (China, Japan, United States)) appear to excel at tasks involving spatial and mathematical reasoning [9, 27], while females excel in verbal fluency and verbal memory and on tasks requiring perceptual speed and fine motor skills [27], These results may be partly due to sex differences in brain structure or girls’ earlier maturation compared with boys. A previous study showed that global white matter volume correlates more strongly with intelligence in women, while global grey matter volume correlates more strongly with intelligence in men [28]. Additionally, cultural (environment) influences should be considered as another possible explanation for the differences in girls and boys’ intellectual functioning. In the present study, girls exhibited a greater prevalence of stunting and underweight, revealing inequalities between boys and girls in rural China from the perspective of girls. Many studies revealed a strong influence of childhood socioeconomic background on intellectual development [29, 30]. In the present study, factors such as household economic status, sex and children’s schooling were strongly associated with children’s intellectual development (data not show). One study revealed that the current general environment is quite complex and stimulating, from pictures on the wall to movies to television to video games to computers, and that each of these items can stimulate children’s cognitive development [31]. Thus, children of higher socioeconomic status, according to parents’ educational level, household economic status and children’s school, could often be exposed to new things and horizons. The lower socioeconomic background of boys and girls in rural China may have resulted in fewer educational opportunities and less access to items that could stimulate their cognitive development. The inequality of socioeconomic background between urban and rural populations in China may also explain the lower WSIC-IV test scores of rural children. Another explanation may be related to the emphasis on girls’ proficiency in household chores and care for younger siblings, resulting in girls having in sufficient time for schoolwork and study [19].

The study findings revealed great sex disparities in rural China. This inequality calls for effective methods to fight against traditional Confucian gender ideology. To decrease gender inequalities in intellectual development, policy makers should focus on the supply, funding and quality of educational materials and health services in rural areas. Furthermore, and the education sector should ensure that rural schools are given priority in the allocation of teaching resources and promote personalised education for the specific cognitive abilities of girls and boys in rural China.

Our study has several limitations and strengths. The limitation of this study is that we did not include all possible confounders (such as children’s diet) in our analysis, mainly because the children display the same dietary habits. The main strength of this study is that information on maternal pregnancy and infants at birth, such as the type of prenatal micronutrient supplementation followed and birth weight, was collected during the time of pregnancy or at birth, ensuring that this study does not suffer the potential biases that may be introduced when using recalled information. In addition, we used well-known, standardised intelligence scales that can be used in different cultures and settings and the latest version of WISC-IV, which could provide more detailed information on the intellectual functioning of 7-10-year-old children. However, the WISC-IV may not be an accurate measure of “intelligence” in its broadest sense, partly because IQ tests examine only particular areas of “intelligence” and fail to account for certain areas that are associated with “intelligence”, such as creativity or emotional intelligence.

## Conclusions

There is no evidence to suggest sex differences in general intelligence in early school-aged children in rural China. However, a difference in general intelligence between 10-year-old boys and girls was evident. Girls and boys in rural China showed different specific cognitive abilities. Excluding the possibility of sex differences in brain structure, inequalities between boys and girls could explain the differences in their performance on intellectual tests. Further research is required to clearly delineate sex differences in intelligence in adolescence and adulthood in rural China. Our findings also emphasise the need for continued efforts to fight against gender inequalities. The education sector should ensure that rural schools are given priority when teaching resources are allocated and encourage the use of different education methods for boys and girls in rural China.

### Availability of data and materials

All relevant data are within the paper and its Supporting Information files.

## Abbreviations

CI: confidence interval
FSIQ: full-scale intelligence quotient
IQ: intelligence quotient
PRI: perceptual reasoning index
PSI: processing speed index
VCI: verbal comprehension index
WISC-IV: Wechsler intelligence scale for children fourth edition
WMI: working memory index
